# Experimental Aerosol Inoculation and Investigation of Potential Lateral Transmission of *Mycobacterium bovis* in Virginia Opossum (*Didelphis virginiana*)

**DOI:** 10.1155/2012/842861

**Published:** 2012-06-03

**Authors:** Karla A. Fenton, Scott D. Fitzgerald, Steve Bolin, John Kaneene, James Sikarskie, Rena Greenwald, Konstantin Lyashchenko

**Affiliations:** ^1^Department of Pathobiology and Diagnostic Investigation Diagnostic Center for Population and Animal Health, College of Veterinary Medicine, Michigan State University, 4125 Beaumont Road, Lansing, MI 48910, USA; ^2^Center for Comparative Epidemiology, College of Veterinary Medicine, Michigan State University, East Lansing, MI 48824, USA; ^3^Department of Small Animal Clinical Sciences, College of Veterinary Medicine, Michigan State University, East Lansing, MI 48824, USA; ^4^Chembio Diagnostic Systems, Inc., 3661 Horseblock Road, Medford, NY 11763, USA

## Abstract

An endemic focus of *Mycobacterium bovis* (*M. bovis*) infection in the state of Michigan has contributed to a regional persistence in the animal population. The objective of this study was to determine if Virginia opossums (*Didelphis virginiana*) contribute to disease persistence by experimentally assessing intraspecies lateral transmission. One wild caught pregnant female opossum bearing 11 joeys (young opossum) and one age-matched joey were obtained for the study. Four joeys were aerosol inoculated with *M. bovis* (inoculated), four joeys were noninoculated (exposed), and four joeys plus the dam were controls. Four replicate groups of one inoculated and one exposed joey were housed together for 45 days commencing 7 days after experimental inoculation. At day 84 opossums were sacrificed. All four inoculated opossums had a positive test band via rapid test, culture positive, and gross/histologic lesions consistent with caseogranulomatous pneumonia. The exposed and control groups were unremarkable on gross, histology, rapid test, and culture. In conclusion, *M. bovis* infection within the inoculated opossums was confirmed by gross pathology, histopathology, bacterial culture, and antibody tests. However, *M. bovis* was not detected in the control and exposed opossums. There was no appreciable lateral transmission of *M. bovis* after aerosol inoculation and 45 days of cohabitation between infected and uninfected opossums.

## 1. Introduction

Numerous wildlife species have proven to be a significant reservoir of *Mycobacterium bovis *(*M. bovis)* some examples include: the Eurasian Badger (*Meles meles*) in Great Britain, the African Buffalo (*Syncerus caffer*) in South Africa, the Brushtail possum (*Trichosurus vulpecula*) in New Zealand, Eurasian wild boar (*Sus scrofa*) in some regions of Spain, and the White-Tailed Deer (*Odocoileus virginianus*) in the United States [1–9]. *Mycobacterium bovis *has the ability to produce disease within a wide range of mammal species including humans, thus making collaborative research, surveillance, and control essential to understanding the epidemiology of this disease. Virginia opossum (*Didelphis virginiana*), family Didelphidae and the Brushtail possum, family Phalangeridae belong to the same order, Marsupialia; however distant, these relatives share similar behavioral traits that may contribute to the spread of tuberculosis [7, 10]. It has been established that Brushtail possums are an ideal host for tuberculosis due to the fact that they are highly susceptible to* M. bovis*, shed the organism through multiple routes and have shared dens [5, 7, 11]. Virginia opossum is a known natural host of tuberculosis in the state of Michigan in the United States and previous studies have shown them to be susceptible to *M. bovis* by aerosol inoculation [5, 6, 12]. Additionally, Virginia opossum utilizes shared dens, and in the state of Michigan, has a high potential for significant interaction with other animals harboring tuberculosis [8]. The aim of the present study was to determine whether the Virginia opossum may contribute to disease spread by characterizing the intraspecies lateral transmission after aerosol inoculation and 45 days of cohabitation. 

## 2. Materials and Methods

### 2.1. Virginia Opossum

One wild caught pregnant female Virginia opossum bearing 11 joeys, approximately 10 weeks old, plus one age matched joey from outside of the litter were obtained. Animals were assessed and clinically judged to be in good health at Michigan State University, College of Veterinary Medicine, Zoo and Wildlife Services. The dam was prophylactically treated with oral fenbendazole (50 mg/kg). Animals were monitored daily and offered a commercially available dry cat food and water ad lib with weekly supplements of granny smith apples or moist canned cat food. Institutional Animal Care and Use Committee (IACUC) approved guidelines were implemented.

The stock *M. bovis* isolate was obtained from the Michigan Department of Community Health (MDCH), Lansing, Michigan, USA, animal 08 TB 883 AF 327 DEER 269398. This pure culture was quantified by plating 100 uL of culture onto Middlebrooks 7H10 agar and incubated at 37°C. The undiluted stock culture was estimated to have 300,000 cfu/mL, aliquots were diluted to the desired concentration of 1 × 10^6^ colony forming units (cfu) per mL [5]. Sedation of the joeys was achieved by intramuscular injection of Telazol (Fort Dodge Animal Health) 100 mg/kg. Four sedated joeys received aerosol inoculation of *M. bovis* (inoculated group), four served as noninoculated in-contact joeys (exposed group), and three joeys, the dam, and the additional age matched joey from outside of the litter served as the control group. *Mycobacterium bovis* was administered to the joeys in the designated inoculated group at a concentration of 1 × 10^6^ cfu via nebulization for a total of 10 minutes [6]. Inoculated joeys were ear-notched for identification purposes. Inoculated and noninoculated (exposed) joeys were housed individually for one week prior to the forty-five days of cohabitation in a BL-3 Horsfall isolator [6]. One noninoculated (exposed) joey was housed with one inoculated joey making four replicate cohabitation groups. The control animals were individually housed in a separate containment room. 

### 2.2. Gross and Histopathology

Individual weight measurements were taken every two weeks until the joeys were sacrificed. At day eighty-four after inoculation or after exposure, joeys were sacrificed by initial sedation with an intramuscular injection of Telazol (100 mg/kg) and subsequent intracardiac exsanguination. Immediately after exsanguination the whole blood samples were clotted at 4°C for 1 hour, centrifuged at 5,000 time gravity for 5 minutes, and serum was then separated into sterile tubes and frozen at −20°C until all samples were collected for the entirety of the study.

A complete postmortem examination was performed. Brain, eye, nasal turbinates, trachea, lungs, heart, liver, kidney, spleen, stomach, pancreas, gonad, adrenal gland, small intestine, large intestine, tonsil, lymph nodes (cranial, thoracic, and abdominal), urinary bladder, skeletal muscle, and pinea were harvested, fixed in 10% neutral-buffered formalin, and trimmed for histopathology. All major organs (lungs, liver, kidney, and spleen) were individually weighed and collected for *M. bovis* culture. Slides were stained with hematoxylin and eosin and Ziehl-Neelsen's acid-fast stain followed by light microscopy examination.

### 2.3. Bacteriology

Tissues were processed for *M. bovis* isolation at Michigan Department of Community Health (MDCH) utilizing standardized protocols [6]. Four tissue groups were pooled for culture. Pool A: cranial lymph nodes and tonsil, Pool B: thoracic lymph nodes and lungs, Pool C: liver, kidney, spleen, abdominal lymph nodes, and Pool D: small intestine and large intestine.

### 2.4. Serology Assay

Serum was sent to a commercial laboratory for rapid test analysis (Chembio Diagnostics Systems Inc., Medford, NY, USA). The rapid test is a lateral-flow, blue latex bead signal-based, qualitative antibody detection assay that utilized a cocktail of selected *M. bovis* antigens (ESAT-6, CFP10, MPB83). The assay uses a ready-to-use plastic cassette containing a nitrocellulose membrane impregnated with the cocktail of test antigens. Thirty microliters of test serum and 3 drops of diluent buffer were added to the test well and the result of the reaction was read by visual evaluation after 20 minutes [13]. An antibody positive sample was indicated by a visible band at both the test and control lines, while an antibody negative sample was indicated by a visible band at the control line but no band at the test line [13].

### 2.5. Statistical Analysis

The two-sample *t*-significance test was calculated on all data sets to determine difference between the inoculated, exposed and control groups. The Student's *t*-test was chosen based on the minimal sample size and distribution of values [14]. The *t*-statistic obtained from the data was compared to the *t* distribution critical values table using the smallest degrees of freedom and *P* value of 0.05 for a one-sided test and 0.025 for a two-sided test [14].

## 3. Results

### 3.1. Gross and Histopathology

All of the joeys gained weight during the extent of the study. The average biweekly weight gain between the three groups of joeys was not remarkably different, inoculated (425 g), exposed (385 g), and controls (502 g), and no significant difference was noted for total body weight gain. Additionally, there was no significant difference noted for any of the major organs across any of the groups. There was no significance noted when comparing total body weight gain of control versus the inoculated opossums, controls versus exposed opossums, and inoculated versus exposed opossums. There was no significance noted when comparing major organ weight of controls versus inoculated opossums for lung, liver, kidney, and spleen. There was no significance noted when comparing major organ weight of controls versus exposed opossums for lung, liver, kidney, and spleen. And finally, there was no significance noted when comparing major organ weight of exposed versus inoculated opossums for lung, liver, kidney, and spleen.

**Figure 1:**
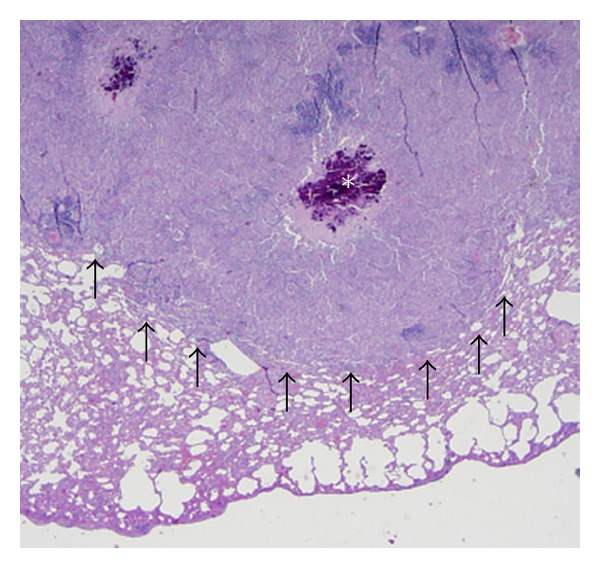
Photomicrograph of a pulmonary tubercle obtained from a *M. bovis* inoculated opossum (2x magnification). Light microscopic features included marked, multifocal, caseogranulomatous pneumonia (outlined by the arrows) with variable amounts of central mineralization (*).

**Figure 2:**
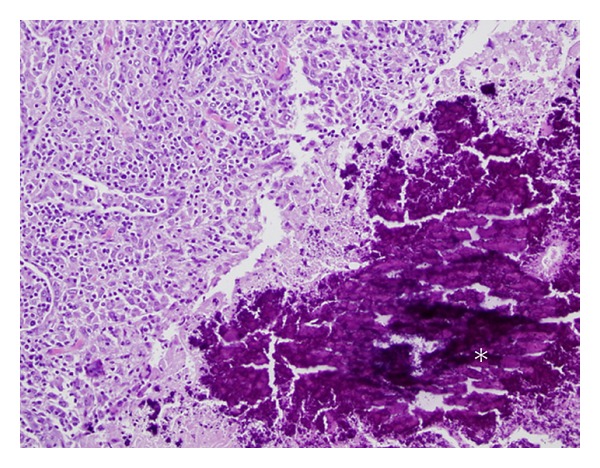
Photomicrograph of a pulmonary tubercle obtained from the opossum described in Figure 1 (40x magnification). Higher magnification of a representative *M. bovis* inoculated opossums characterized by marked, multifocal, caseogranulomatous pneumonia with variable amounts of central mineralization (*).

On gross examination, the lungs of all four inoculated opossums were characterized by marked multifocal to coalescing, raised, white, firm caseogranulomatous nodules distributed throughout all lung lobes which on histological examination were characterized by multifocal caseogranulomatous pneumonia (Figures 1 and 2). No gross or histologic lesions were noted in the exposed or control opossums.

### 3.2. Bacteriology

The isolation of *M. bovis *from pulmonary tissue was successful in all the inoculated opossums. *M. bovis* was isolated from pooled samples of liver, kidney, and spleen in half of the inoculated group (see Table 1). Bacterial cultures for *M. bovis* were negative for all control and exposed opossums.

### 3.3. Serology

**Figure 3:**
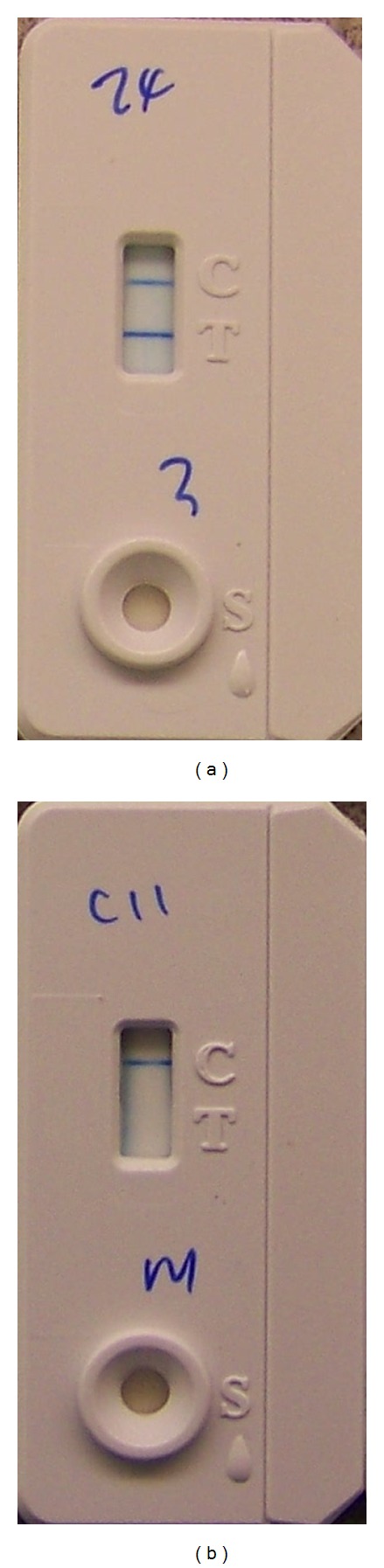
Representative results for the rapid test. (a) The cassette to the left displays a positive band at the control (C) and test (T) window, representing a positive *M. bovis* result. (b) The cassette to the right only displays a positive band at the control (C) window and no band at the test (T) window, representing a negative *M. bovis* result.

 The rapid test identified positive results in all of the inoculated opossums. The exposed and control opossums were rapid test uniformly negative (Figure 3).

## 4. Discussion

This study investigated the potential for intraspecies lateral transmission of *M. bovis* in Virginia opossum. Part of the justification for this investigation was the well-established role of the Brushtail possum, a distant relative of the Virginia opossum, as a reservoir host of *M. bovis* and their role in the epidemiology of animal tuberculosis in New Zealand [7, 11]. There is little information on the potential of the Virginia opossum population as a reservoir of *M. bovis* or spread of infection within the population [5, 6].

This study failed to demonstrate any detectable horizontal transmission from opossums infected by aerosol with *M. bovis* to exposed opossums. All of the inoculated animals had gross, histologic, bacterial culture, and serologic positive tests for tuberculosis, whereas the exposed and control groups had no gross or histologic lesions and remained serologically negative. Typical gross and histologic lesions of multifocal caseogranulmatous pneumonia were noted within all of the inoculated opossums. All inoculated opossums were culture positive for *M.bovis* from the respiratory tissue (pooled thoracic lymph nodes and lung) and half of these were also positive from systemic tissue (pooled liver, kidney, spleen, and abdominal lymph nodes). By day 84 after inoculation, the disease was widely disseminated in half of the inoculated opossums but these opossums did not show any clinical signs of illness, emaciation, or draining tracts. This is in contrast to the Brushtail possums with natural *M. bovis* infection, where the disease is highly progressive and fatal. The mean survival time of Brushtail possums with natural tuberculous is 4.7–14 months and with experimental tuberculosis is 8 weeks after inoculation by intratracheal inoculation [4, 5, 11]. The present study did not address the clinical manifestations of chronic disease progression or bacterial shedding in Virginia opossums; this should be investigated in future studies.

Recent advances in development of serologic assays for antemortem detection of *M. bovis *infection in multiple-host species include the Chembio rapid test [13]. In the present study, this serodiagnostic method was able to identify all inoculated opossums as positive and the exposed and control opossums as negative. Interestingly, two of the four infected animals in which *M. bovis *cultures were isolated from both the respiratory tissues and the systemic tissues showed very prominent test bands on the rapid test. This observation suggests that the disease burden may be associated with antibody levels; further assessment of disease burden in the context of the infectious dose should be addressed in future studies to fully characterize this potential association. The intradermal tuberculin test is a traditional method that is often performed to determine tuberculosis status in live animals [3, 8, 12, 15, 16]. In our experiment we attempted to evaluate intradermal pinnal injections of bovine purified protein derivative (bPPD) but this procedure was difficult and was subsequently discontinued (data not shown).

In conclusion, experimental *M. bovis* aerosol infection of Virginia opossums produced pathological, bacteriological, and serological evidence of tuberculosis. However, *M. bovis *was not detected in exposed opossums after 45 days of cohabitation between aerosol-infected suggesting no appreciable lateral transmission of *M. bovis*. Future studies may be warranted to assess shedding patterns and chronic disease progression in Virginia opossum involving a longer exposure period.

## Figures and Tables

**Table 1:** *Mycobacterium bovis* culture group results. The column to the left indicates the opossum group as *M. bovis* inoculated, exposed, or control. The first row indicates the four pools that were created for culture. Pool A: cranial lymph nodes and tonsil, Pool B: thoracic lymph nodes, Pool C: liver, kidney, spleen, and abdominal lymph nodes, and Pool D: small intestine and large intestine. The body of the table is split into boxes indicating the positive (Pos) and negative (Neg) *M. bovis* culture results.

Inoculation group	Pool A upper respiratory		Pool B lower respiratory		Pool C systemic		Pool D alimentary	
	Pos	Neg	Pos	Neg	Pos	Neg	Pos	Neg
Inoculated	0	4	4	4	2	4	0	4
Exposed	0	4	0	4	0	4	0	4
Controls	0	4	0	4	0	4	0	4
